# Network Pharmacology–Based Prediction and Pharmacological Validation of Effects of Astragali Radix on Acetaminophen-Induced Liver Injury

**DOI:** 10.3389/fmed.2022.697644

**Published:** 2022-07-04

**Authors:** Yuan Peng, Gerui Zhu, Yuanyuan Ma, Kai Huang, Gaofeng Chen, Chenghai Liu, Yanyan Tao

**Affiliations:** ^1^Institute of Liver Diseases, Shuguang Hospital Affiliated to Shanghai University of Traditional Chinese Medicine, Shanghai, China; ^2^Shanghai Key Laboratory of Traditional Chinese Clinical Medicine, Shanghai, China; ^3^Key Laboratory of Liver and Kidney Diseases, Ministry of Education, Shanghai, China

**Keywords:** Astragali Radix, network pharmacology, acetaminophen, acetaminophen-induced acute liver injury, hepatocytes

## Abstract

Astragali Radix (AR) has been widely used in traditional Chinese medicine prescriptions for acute and chronic liver injury. However, little is known about the effects of AR on acetaminophen (APAP)-induced liver injury (ALI). In the current study, a network pharmacology–based approach was applied to characterize the action mechanism of AR on ALI. All compounds of AR were obtained from the corresponding databases, and active compounds were selected according to its oral bioavailability and drug-likeness index. The potential genes of AR were obtained from the Traditional Chinese Medicine Systems Pharmacology Database and Analysis Platform (TCMSP), and the Bioinformatics Analysis Tool for Molecular Mechanism of Traditional Chinese Medicine (BATMAN-TCM) and PubChem, whereas the potential genes related to ALI were obtained from Online databases (GeneCards and Online Mendelian Inheritance in Man) and Gene Expression Omnibus profiles. The enriched processes, pathways, and target genes of the diseases were analyzed by referring to the Search Tool for the Retrieval of Interacting Genes/Proteins database. A network constructed through Cytoscape software was used to identify the target proteins that connected the compounds in AR with the differential genes of ALI. Subsequently, the potential underlying action mechanisms of AR on ALI predicted by the network pharmacology analyses were experimentally validated in APAP-induced liver injury in mice and HL7702 cells incubated with APAP. The compound-target network included 181 targets, whereas the potential genes related to ALI were 4,621. A total of 49 AR–ALI crossover proteins, corresponding to 49 genes, were filtered into a protein–protein interaction network complex and designated as the potential targets of AR on ALI. Among the genes, the three highest-scoring genes, *MYC*, *MAPK8*, and *CXCL8* were highly associated with apoptosis in ALI. Then *in vitro* and *in vivo* experiments confirmed that AR exhibited its prominent therapeutic effects on ALI mainly via regulating hepatocyte apoptosis related to inhibiting the expressions of *MYC* (c-Myc), *MAPK8* (JNK1), and *CXCL8* (IL-8). In conclusion, our study suggested that the combination of network pharmacology prediction with experimental validation might offer a useful tool to characterize the molecular mechanism of AR on ALI.

## Introduction

Acetaminophen (*N*-acetyl-*p*-aminophenol, APAP) is a widely utilized over-the-counter medication for relief of fever and pain and it has a dose-related toxic effect. APAP toxicity accounts for above 40% of acute liver failure cases in the United States, Great Britain, and Europe (1). APAP is safe at recommended doses, 85–90% of APAP is metabolized into non-toxic products excreted into the urine, 2% is excreted into the urine unchanged, and <10% is metabolized by the cytochrome P450 into the reactive metabolite *N*-acetyl-*p*-benzoquinone imine (NAPQI). NAPQI is rapidly converted to non-toxic products by glutathione (GSH) (2). However, once APAP is overdosed, excessive NAPQI will be formed, which depletes hepatic GSH stores. Subsequently, the remaining NAPQI will react with the cellular proteins and cause oxidative stress, microsomal membrane damage, and even cell death (3), which leads to severe hepatotoxicity and even liver failure (4, 5).

*N*-acetylcysteine (NAC) is widely used in patients with APAP-induced liver injury (ALI). The mechanism of NAC involves maintaining intracellular GSH storage and detoxifying NAPQI in the early stages of ALI (6). In fact, the highest peak of serum transaminase activities usually occurs 48–96 h after an acute over dose of APAP ingestion. In the case of undetectable transaminase levels, the patient may present in liver failure days after ingestion. However, the effects of NAC on ALI weaken over time. In addition, intravenous NAC can lead to intravenous urticaria, itching, and other adverse reactions (7). Therefore, it is of great significance to find more alternative drugs for the treatment of APAP-induced ALI. Astragali Radix (AR, Huangqi in Chinese), a popular Chinese Herb Medicine and dietary supplement, has more than 2,000 years of history for medicinal use (8). Clinically, Astragali Radix and its recipe as the main drug can be used to treat acute and chronic liver diseases such as liver injury, non-alcoholic fatty liver disease (NAFLD) or liver fibrosis (9–11). AR has also been proved to be a tonic with antioxidant effects for oxidative damage of brain induced by doxorubicin (12). However, it is unclear whether AR has the effect of treating ALI, let alone its mechanism. Our results might add new dimension to the hepato-protective effect and mechanism of AR, which further expanded the clinical application of AR in ALI.

**FIGURE 1:**
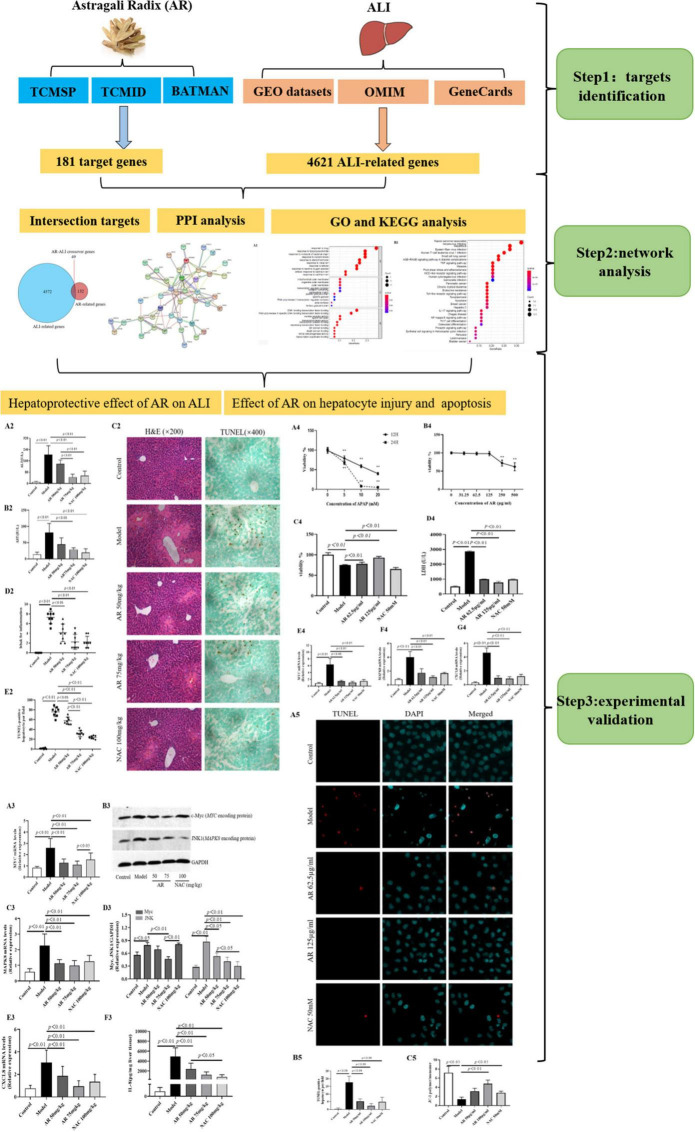
Flowchart of the network pharmacological and experimental studies of Astragali Radix (AR) in APAP-induced acute liver injury (ALI).

We screened online databases for target genes of the compounds in AR and genes related to ALI in patients. A network pharmacology approach was used to analyze the enriched processes, pathways, and diseases related to the target genes. Then, *in vivo* and *in vitro* experimental assays were performed to validate the effects of AR on ALI and their underlying mechanism. Figure 1 displays the workflow of our network pharmacology approach and the experiments conducted to determine whether AR can inhibit ALI and the possible mechanism involved. The preventative effects of AR against ALI were predicted using the network pharmacology approach, and the mechanism associated with these effects involves inhibiting hepatocyte apoptosis *in vivo* and *in vitro*. These results may represent a breakthrough in the potential use of AR in the design of strong anti-ALI drugs.

## Materials and Methods

### Ethics

All experimental procedures complied with the Guidelines for Experimentation of Shuguang Hospital, affiliated with Shanghai University of Traditional Chinese Medicine. The protocols were reviewed and approved by the Ethics Committee of the institution.

### Databases

Data on the canonical name and molecular weight of the compounds in AR were obtained from the Traditional Chinese Medicine Systems Pharmacology Database and Analysis Platform (TCMSP^1^ ; version 2.3, updated on April 22, 2020) (13), the Bioinformatics Analysis Tool for Molecular Mechanism of Traditional Chinese Medicine (BATMAN-TCM^2^ ; updated on April 22, 2020 (14), and PubChem^3^ (updated on April 22, 2020) (15). To identify the compounds with high levels of oral absorption, usability, and biological activity for further study, candidate components were screened and selected based on the following two parameters: (1) oral bioavailability (OB) of ≥30% and (2) drug likeness (DL) of ≥0.18 (Table 1).

**TABLE 1:** Components in Astragali Radix (AR).

Mol ID	Molecule Name	MW	OB (%)	DL
MOL000211	Mairin	456.78	55.38	0.78
MOL000239	Jaranol	314.31	50.83	0.29
MOL000296	Hederagenin	414.79	36.91	0.75
MOL000033	(3S,8S,9S,10R,13R,14S,17R)-10,13-dimethyl-17-[(2R,5S)-5-propan-2-yloctan-2-yl]-2,3,4,7,8,9,11,12,14,15,16,17-dodecahydro-1H-cyclopenta[a]phenanthren-3-ol	428.82	36.23	0.78
MOL000354	Isorhamnetin	316.28	49.6	0.31
MOL000371	3,9-Di-*O*-methylnissolin	314.36	53.74	0.48
MOL000374	5′-Hydroxyiso-muronulatol-2′,5′-di-*O*-glucoside	642.67	41.72	0.69
MOL000378	7-*O*-Methylisomucronulatol	316.38	74.69	0.3
MOL000379	9,10-Dimethoxypterocarpan-3-*O*-β-D-glucoside	462.49	36.74	0.92
MOL000380	(6aR,11aR)-9,10-Dimethoxy-6a,11a-dihydro-6H-benzofurano[3,2-c]chromen-3-ol	300.33	64.26	0.42
MOL000387	Bifendate	418.38	31.1	0.67
MOL000392	Formononetin	268.28	69.67	0.21
MOL000398	Isoflavanone	316.33	109.99	0.3
MOL000417	Calycosin	284.28	47.75	0.24
MOL000422	Kaempferol	286.25	41.88	0.24
MOL000433	FA	441.45	68.96	0.71
MOL000438	(3R)-3-(2-Hydroxy-3,4-dimethoxyphenyl)chroman-7-ol	302.35	67.67	0.26
MOL000439	Isomucronulatol-7,2′-di-*O*-glucosiole	626.67	49.28	0.62
MOL000442	1,7-Dihydroxy-3,9-dimethoxy pterocarpene	314.31	39.05	0.48
MOL000098	Quercetin	302.25	46.43	0.28

### Prediction of Drug-Related Targets of Bioactive Components

The two databases, TCMSP and BATMAN-TCM, were used to predict the target genes of the traditional Chinese medicine (TCM) ingredients (13, 14). The known therapeutic targets of these ingredients were confirmed by referring to the DrugBank database^4^ (updated on February 28, 2020) and designated as the putative targets of AR. The putative targets were designated as the drug-related genes.

### Prediction of Acetaminophen-Induced Liver Injury-Related Genes From Gene Expression Omnibus Profiles

Gene expression microarray data (GSE74000) of liver samples from patients suffering from APAP-induced ALI and from healthy livers were obtained from the Gene Expression Omnibus (GEO) database of the National Center for Biotechnology Information^5^, a public functional genomics data repository. Data on two healthy liver samples (GSM1907918 and GSM1907919) and three APAP-induced ALI liver samples (GSM1907915, GSM1907916, and GSM1907917) were obtained. By using the data from the whole human genome microarray, we analyzed the potential therapeutic targets for patients with and without ALI by employing the LIMMA package in the R language. An adjusted *p* value of <0.05 and a fold change (FC) of >1.2 were selected as the cutoff criteria. The potential genes were considered ALI-related target genes and processed by using Strawberry Perl (version 5.30.0.1) and R (version 4.0.2) with the Bioconductor packages.

### Prediction of Acetaminophen-Induced Liver Injury-Related Targets From Online Databases

Information regarding the various genes associated with ALI were obtained from the GeneCards database^6^ and the Online Mendelian Inheritance in Man (OMIM) database^7^, which are knowledge bases for data on human genes and genetic disorders (16, 17). The key search terms used to search the two databases were “acetaminophen-induced liver injury” and “acute APAP-induced liver injury,” and the results were exported online.

### Processing of Data on Potential Hub Genes Involved in Effects of Astragali Radix on Acetaminophen-Induced Liver Injury

The predictions of the ALI-related genes and putative drug-related genes were verified using the VennDiagram R package. Overlapping genes were considered potential hub genes related to the preventative effects of AR against ALI (Table 2).

**TABLE 2:** The potential hub genes of Astragali Radix (AR) against acetaminophen-induced liver injury (ALI).

Gene	Full name
ABCG2	ATP binding cassette subfamily G member 2
ADH1B	Alcohol dehydrogenase 1B (class I), beta polypeptide
ADH1C	Alcohol dehydrogenase 1C (class I), gamma polypeptide
AHR	Aryl hydrocarbon receptor
AKR1C3	Aldo-keto reductase family 1 member C3
BAX	*BCL2 associated X, apoptosis regulator*
BCL2	BCL2 apoptosis regulator
BCL2L1	*BCL2L1 – BCL2 like 1*
CCND1	*Cyclin D1*
CHRM2	Cholinergic receptor muscarinic 2
CHUK	Component of inhibitor of nuclear factor kappa B kinase complex
COL1A1	*Collagen type I alpha 1 chain*
CTSD	*Cathepsin D*
CXCL2	*C-X-C motif chemokine ligand 2*
CXCL8	*C-X-C motif chemokine ligand 8*
CYP1A2	*Cytochrome P450 family 1 subfamily A member 2*
CYP3A4	*Cytochrome P450 family 3 subfamily A member 4*
DIO1	*Iodothyronine deiodinase 1*
E2F1	*E2F transcription factor 1*
E2F2	*E2F transcription factor 2*
F7	*Coagulation factor VII*
FOS	*Fos proto-oncogene, AP-1 transcription factor subunit*
ICAM1	*Intercellular adhesion molecule 1*
IRF1	*Interferon regulatory factor 1*
LYZ	*Lysozyme*
MAOB	*Monoamine oxidase B*
MAPK14	*Mitogen-activated protein kinase 14*
MAPK8	*Mitogen-activated protein kinase 8*
MYC	*MYC proto-oncogene, bHLH transcription factor*
NCF1	*Neutrophil cytosolic factor 1*
NCOA1	*Nuclear receptor coactivator 1*
NCOA2	*Nuclear receptor coactivator 2*
ND6	*NADH dehydrogenase subunit 6*
NFE2L2	*Nuclear factor, erythroid 2 like 2*
NFKBIA	*NFKB inhibitor alpha*
NOS2	*Nitric oxide synthase 2*
NR1I2	*Nuclear receptor subfamily 1 group I member 2*
NR1I3	*Nuclear receptor subfamily 1 group I member 3*
ODC1	*Ornithine decarboxylase 1*
PGR	*Progesterone receptor*
PON1	*Paraoxonase 1*
PPARD	*Peroxisome proliferator activated receptor delta*
SELE	*Selectin E*
SLPI	*Secretory leukocyte peptidase inhibitor*
SOD1	*Superoxide dismutase 1*
SPP1	*Secreted phosphoprotein 1*
STAT1	*Signal transducer and activator of transcription 1*
TOP1	*DNA topoisomerase I*
TOP2A	*DNA topoisomerase II alpha*

### Network Construction and Analysis

On the basis of the potential hub genes identified through the aforementioned process, a protein–protein interaction (PPI) network was constructed using the public Search Tool for the Retrieval of Interacting Genes/Proteins database^8^ (updated May 2, 2020) (18). The minimum required interaction score was set to 0.7 to improve the accuracy of the results. For further observations of the biological functions of the hub genes, the clusterProfiler package was used to explore and visualize the data used in the pathway and functional enrichment analysis. Cytoscape (version 3.7.1) was used to construct and visualize a network of the compound–target protein-differential genes. This network can be used to identify the target proteins that connect compounds in AR with differential genes.

### Gene Ontology and Kyoto Encyclopedia of Genes and Genomes Pathway Analysis

To identify the functional annotation and pathway enrichment associated with the potential genes, a pathway enrichment analysis was performed on data from the Gene Ontology (GO) annotation database^9^ and the Kyoto Encyclopedia of Genes and Genomes (KEGG)^10^ by using the clusterProfiler R package (19, 20). The enrichplot and DOSE Bioconductor packages were used to visualize the results of the enrichment and facilitate the interpretation process (21). Biological process (BP), molecular function (MF), and cellular component (CC) were used to visualize the functional annotation and pathway of the potential genes. Enrichment with a *p* value of <0.05 was considered statistically significant, and an adjusted *p* value of <0.05 was set as the threshold value.

### Reagents

BioReagent-grade NAC (Cat No. A7250) and APAP (Cat No. 908312) were obtained from Sigma-Aldrich (Shanghai, China). All other chemicals and solvents were of analytical grade.

### Drug Preparation and Identification

**FIGURE 2:**
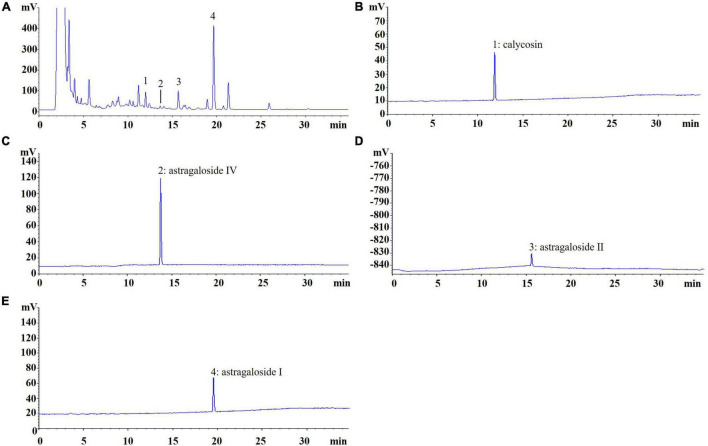
Chromatogram of Astragali Radix (AR). **(A)** The peak No. refer to standard content, **(B)** 1: calycosin (0.1129 mg/g); **(C)** 2: astragaloside IV (0.0037 mg/g); **(D)** 3: astragaloside II (0.5141 mg/g); **(E)** 4: astragaloside I (1.4641 mg/g).

AR was purchased from Shanghai Shyndec Pharmaceutical Co., Ltd. (Shanghai, China). The AR extract was prepared as follows: dried AR (1,000 g) was extracted with 10 L of 70% ethanol (v/v) twice in reflux for 1 h each time. Extracts were combined, filtered, and concentrated under reduced pressure at 50°C and dried through lyophilization to yield 240 g of AR extract. A quantitative analysis of the active compounds was performed through high-performance liquid chromatography with an evaporative light scattering detector in an Agilent 1100 system (Santa Clara, CA, United States). The Agilent 1100 ChemStation software was used for the data analysis, and Figure 2 displays the results.

### Mouse Models

Male C57BL/6 mice weighing 25 ± 2 g were supplied by Beijing Vital River Laboratory Animal Technology Co., Ltd. [License No.: SCXK(Jing)2014-0001]. The mice were fed in the Experimental Animal Center of Shanghai University of Traditional Chinese Medicine (License No: SCXK[Hu]2020-0009) and were acclimatized to the animal center conditions for 5 days before the experiments. The mice were given Rodent Laboratory Chow and water *ad libitum* and maintained under controlled conditions with a temperature of 22°C–25°C, relative humidity of 46–52%, and a 12/12-h light/dark cycle (lights on at 7:00 a.m.). Our experiments were conducted in accordance with the Animal Ethics Committee of Shanghai University of Traditional Chinese Medicine.

### Induction of Acetaminophen-Induced Liver Injury *in vivo*

The mice were experimentally induced with ALI. APAP was dissolved in 1% carboxymethylcellulose sodium in water, and the mice were intragastrically administered the mixture at 350 mg/kg body weight (22, 23). The mice were anesthetized with pentobarbital sodium and euthanized 12 h after APAP administration. Blood samples were collected from the inferior vena cava and centrifuged at 3,000 rpm for 30 min at 4°C after 3 h, and the serum was maintained at −70°C for subsequent liver function tests. The livers were promptly removed, and liver tissue was taken from the right lobe of the livers, fixed in 10% phosphate-buffered formaldehyde, and processed for paraffin embedding.

### Animal Groups and Experimental Design

To evaluate the effect of AR on ALI, the mice were randomly divided into five groups: control (*n* = 7), model (APAP 350 mg/kg; *n* = 8), AR 50 mg/kg (*n* = 8), AR 75 mg/kg (*n* = 8), and NAC (*n* = 8) groups. The mice in the two AR groups were intragastrically administered at doses of 50 and 75 mg/kg body weight once a day for three consecutive days, respectively. Meanwhile, the mice in NAC group were intragastrically administered with NAC (100 mg/kg), and mice in the control and model groups received an equal volume of distilled water. On the 4th day, except the control group, the mice in the other groups were intragastrically administered with 350 mg/kg APAP. All mice were sacrificed 12 h after APAP administration.

### Serum Levels of Liver Function

Serum alanine aminotransferase (ALT) and aspartate aminotransferase (AST) levels were measured using the BIO-TEK/MQX200R SpectraMax-M5 Multifunctional microplate reader (Molecular Devices, Inc., Sunnyvale, CA, United States) in accordance with the manufacturer’s instructions. Liver function test kits were purchased from Nanjing Jiancheng Bioengineering Institute (Nanjing, China).

### Hepatic Histopathology

To observe the pathological changes of the liver, tissues were fixed in a 10% formaldehyde solution, embedded in paraffin, and cut into 4-μm-thick slices. The tissue slices were subjected to hematoxylin and eosin staining (Nanjing Jiancheng Bioengineering Institute) in accordance with procedures described elsewhere (24). The images were analyzed using a light microscope (Olympus BX40, Japan). Semi-quantification of liver inflammation and liver tissue necrosis was performed by a blinded liver pathologist at 200× and graded along the Knodell scoring system (25).

### Enzyme-Linked Immunosorbent Assay

Liver homogenates were obtained by homogenizing in 50 mM PBS buffer (pH 7.4) in ice water bath and centrifuging at 10,000 × g for 15 min at 4°C for ELISA analysis. Level of IL-8 (*CXCL8* encoding protein) was measured in liver homogenates using commercially available enzyme-linked immunosorbent assay (ELISA) kit for mouse cytokines (F10850, Xitang Bio-Technology Co., Ltd., Shanghai, China). For each assay, liver homogenates of all mice were serially diluted to ensure that values obtained were within the linear range of the standards provided with each kit. The manufacturer’s instructions were strictly followed during the ELISA experiments.

### Western Blot Analysis

Liver tissues were homogenized in RIPA lysis buffer (50 mM Tris-HCl pH 7.4, 150 mM NaCl, 0.1%SDS, 1% Non-idet P-40, 1 mM EDTA, 1 mM PMSF, 1x Roche complete mini protease inhibitor cocktail). The supernatants were collected after centrifugation at 10,000 g at 4°C for 15 min. Protein concentration was determined using a BCA protein assay kit. Equal amount of proteins were separated by 10% SDS gel electrophoresis (SDS-PAGE) under denaturing condition, and then transferred to nitrocellulose membrane. The membrane was blocked with 5% non-fat milk in TBST at room temperature for 1 h, and incubated with primary antibody [c-Myc (ab32072, 1:500), JNK1 (*MAPK8* encoding protein, ab110724, 1:500) and GAPDH (ab205718, 1:2000)] at 4°C overnight. After washing in TBST, the blots were incubated with horseradish-coupled secondary antibody (ab205718). The signals were visualized using the enhance system (ECL).

### Terminal Deoxyuridine Triphosphate Nick-End Labeling Staining

To detect hepatocellular apoptosis, the paraffin-embedded sections were stained through terminal deoxyuridine triphosphate nick-end labeling (TUNEL) staining in accordance with the manufacturer’s protocols (ab206386, Abcam, United States). The images were scanned using an Olympus BX40. A total of five fields under 400× magnification was randomly selected for counting the apoptotic hepatocytes on each slide, and the results are expressed as the number of apoptotic hepatocytes per field.

### Cell Culture

The human hepatic cell line HL7702 (L-02), an immortal cell line derived from human adult hepatic tissue, was purchased from the Shanghai Institute of Biochemistry and Cell Biology of the Chinese Academy of Sciences (Shanghai, China) and was maintained in 1640 Dulbecco’s modified Eagle’s medium supplemented with 10% fetal bovine serum, 100 units/mL penicillin, 100 μg/mL streptomycin, and 2 mM glutamine. The cells were grown in a humidified incubator at 37°C under a 5% CO_2_ atmosphere.

### Drug Incubation

Astragali Radix was dissolved as a concentrated stock solution in dimethylsulfoxide (Sigma-Aldrich, D2660, United States). To determine the incubation time and concentration of APAP, the L-02 cells were cultured with 5, 10, or 20 mM APAP for 12 or 24 h. Subsequently, 20 mM APAP was selected to incubate the L-02 cells for 24 h based on cell viability. The cells were kept in serum-free medium or treated with the following: (1) 20 mM APAP, (2) 20 mM APAP and 62.5 μg/mL AR, (3) 20 mM APAP and 125 μg/mL AR, or (4) 20 mM APAP and 50 mM NAC for 12 h.

### Cell Viability Assay

Cell viability assays were performed using the Cell Counting Kit 8 (MedChemExpress, HY-K0301, China) in accordance with the manufacturer’s protocol.

### Lactate Dehydrogenase Assay

The L-02 cells were seeded at a density of 5,000 cells per well in 96-well plates. After the cells had grown for 24 h under normal growth conditions, the medium was replaced with 1,640 medium containing APAP, APAP and AR, or APAP and NAC. The cells were incubated for another 24 h. The activity of lactate dehydrogenase (LDH) in the cell culture medium (extracellular LDH) was determined by using a commercial LDH kit in accordance with the manufacturer’s instructions.

### Immunofluorescence Staining for JC-1

The L-02 cells cultured in the 96 wells were washed with cold phosphate-buffered saline (PBS) twice and fixed with cold methanol:acetone (1:1) for 10 min on ice. After extensive washing with PBS three times, the cells were permeated with 0.05% saponin for 15 min. Mitochondrial transmembrane potential was assayed through immunofluorescence staining of JC-1 (Life Technologies, T3168, United States), and cells were double-stained with DAPI (Life, C0038, United States) to visualize the nuclei in accordance with the manufacturer’s protocol. Images were taken using an Olympus-FV10l confocal system (Japan).

### Real-Time Fluorescence Quantitative Polymerase Chain Reaction

Total RNA was extracted using TRIzol Reagent (Sangon Biotech, Shanghai, China) in accordance with the manufacturer’s protocol. After the concentration of RNA was determined, 500 ng of total RNA was used as a template for reverse transcription into single-stranded cDNA with the random primers and reagents contained in the Reverse Transcription Reagent Kit with gDNA Eraser (Takara, Dalian, China). Real-time fluorescence quantitative polymerase chain reaction was performed using SYBR Premix Ex Taq (Tli RNaseH Plus; Takara) and the ViiA 7 Real-Time PCR System (ABI, Carlsbad, CA, United States). The β-actin gene was amplified as an internal control, and the primers, along with their sequences, are listed in Table 3. The relative gene quantities compared with the expression levels of β-actin were calculated through the 2^–ΔΔCT^ method.

**TABLE 3:** Real-time quantitative PCR primers used in this study.

Gene	Forward (5′→3′)	Reverse (5′→3′)
MYC	CGACGAGACCTTCATCAAAAAC	CTTCTCTGAGACGAGCTTGG
MAPK8	ACACCACAGAAATCCCTAGAAG	CACAGCATCTGATAGAGAAGGT
CXCL8	AAGGTGAATGGCTGGATTTTTG	CCCAGATGCTGAGACATATGAA
β-Actin	TGA CGA GGC CCA GAG CAA GA	ATG GGC ACA GTG TGG GTG AC

### Statistical Analysis

All data were analyzed using the PASW Statistics software (version 18). Differences between the groups were assessed through a non-parametric one-way analysis of variance. Values are presented as mean ± standard deviation. A *p* value of <0.05 was considered statistically significant.

## Results

### Putative Ingredients and Targets for Astragali Radix

We selected a total of 20 ingredients on the basis of the following criteria for the absorption, distribution, metabolism, and excretion (ADME) properties of drugs with potential biological effects at a systematic level: OB ≥ 30% and DL ≥ 0.18 (Table 1). The potential targets of AR were predicted by referring to the TCMSP, BATMAN-TCM and DrugBank databases, as described in the “Materials and Methods” section. A total of 181 potential target genes for AR were identified (Supplementary Table 1) after the redundant data were removed.

### Differentially Expressed mRNAs in Patients With and Without Acetaminophen-Induced Liver Injury

**FIGURE 3:**
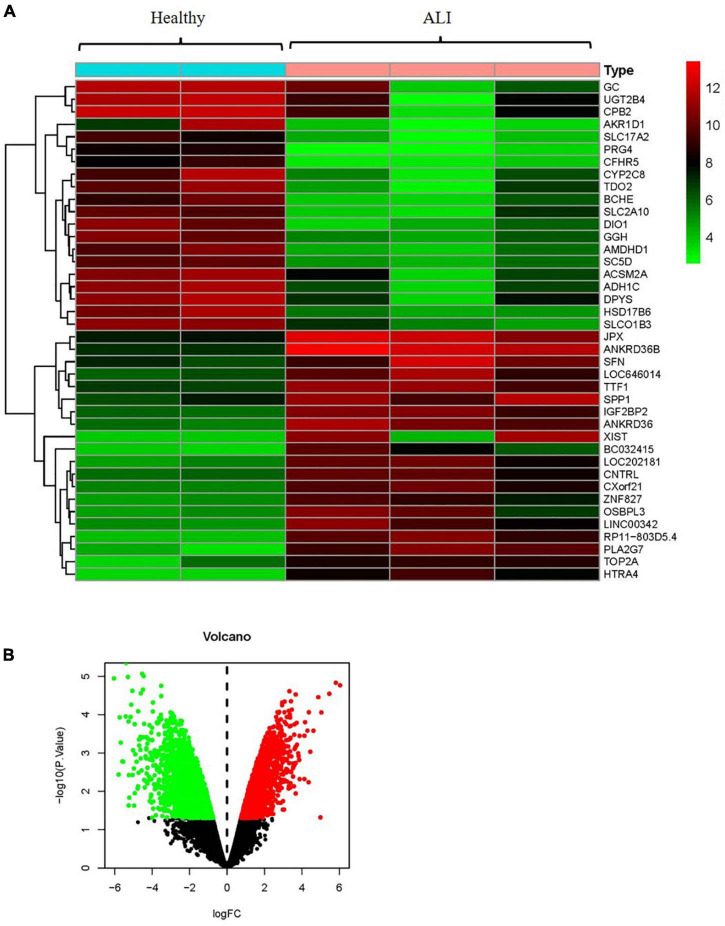
Analyses of the different potential therapeutic targets between ALI and healthy liver tissues. **(A)** The heatmap comparing the different gene expressions between ALI and healthy liver tissues was shown. **(B)** The volcano plot of *P* values as a function of weighted fold change for mRNAs in ALI and healthy liver tissues. The vertical dotted line delimits up- and down-regulation. Red and green plots represent significant up-regulated and down-regulated mRNAs with >1.2-fold change and corrected *P* < 0.05, respectively. ALI, acute liver injury.

By using the raw data from the GSE74000 dataset, we analyzed mRNA expression profiles in the liver biopsy samples from patients with and without ALI. Figure 3A presents a heatmap of the expression of the top 40 genes in the liver samples from the patients with ALI and the controls. Volcano plot filtering identified 4,337 differentially expressed mRNAs (*p* < 0.05, FC > 1.2, Figure 3B). Among them, 1984 mRNAs were upregulated, and 2,353 mRNAs were downregulated. Supplementary Table 2 presents the detailed information regarding these therapeutic targets used in the data analysis. In addition, the ALI-related 284 targets were predicted using the GeneCards and OMIM online databases (Supplementary Tables 3, 4).

### Construction of Astragali Radix Target–Acetaminophen-Induced Liver Injury Network

**FIGURE 4:**
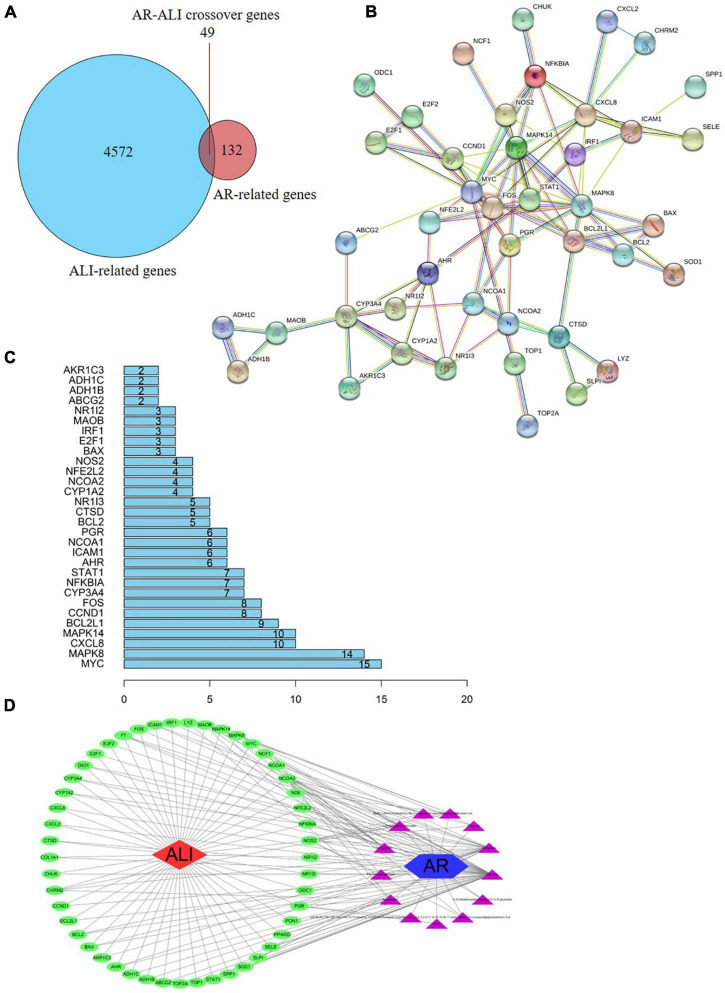
Network analysis of targets. **(A)** Venn diagram of AR- and ALI-related proteins. The overlapped genes were considered as the potential hub genes of AR against ALI. **(B)** Cluster analysis of the PPI network. 49 AR-ALI crossover proteins were filtered into the PPI network complex. **(C)** Histogram of key proteins. The y-axis represents the name of genes, the x-axis represents the number of adjacent genes, and height is the number of gene connections. **(D)** The Component-Target protein network. The triangles represent the 13 candidate active compounds in AR. The green circles represent the gene names of target proteins of ALI found by screening from GEO and online database. ALI, acute liver injury; AR, astragali radix; PPI, protein–protein interaction; GEO, Gene Expression Omnibus.

Based on the identified AR- and ALI-related target genes, a Venn diagram of the AR- and ALI-related proteins was created. The overlapping genes were considered differentially expressed genes (DEGs) and designated as potential hub genes for the effects of AR on ALI (Figure 4A). A total of 49 DEGs (i.e., the overlapping AR–ALI proteins), such as *MYC, MAPK8, CXCL8, MAPK14*, and *BCL2L1*, were filtered into the PPI network complex and presumed to be related to the effects of AR on ALI (Figures 4B,C). In addition, a PPI network of the 49 DEGs was constructed (Figure 4B). To identify the mechanism underlying the effects of AR on ALI, an AR target–ALI network was constructed (Figure 4D).

**FIGURE 5:**
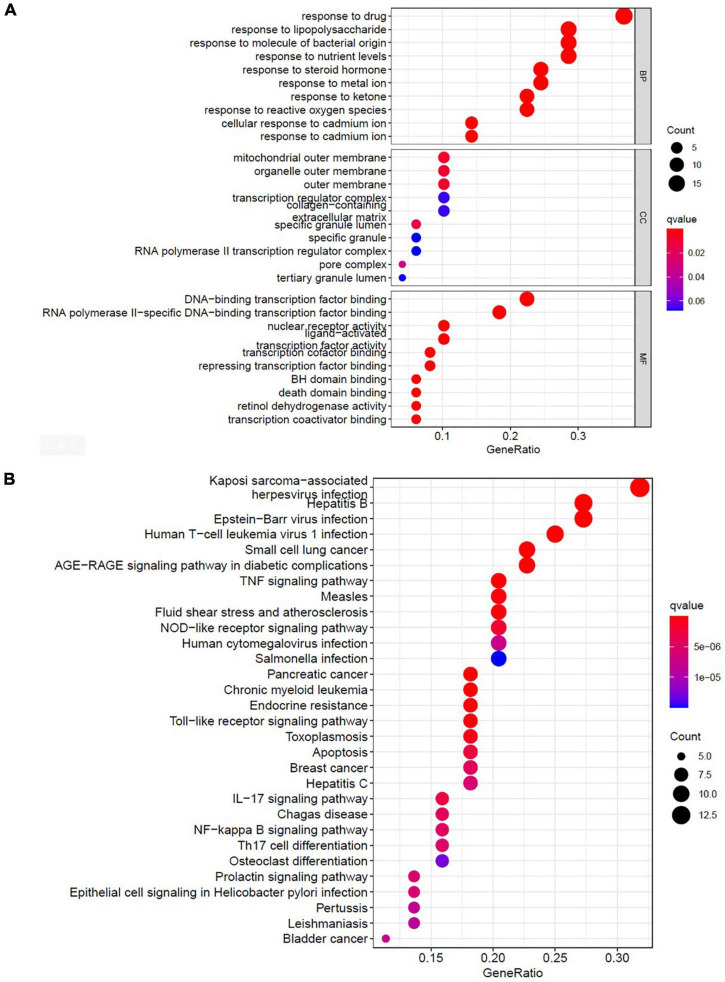
Bioinformatic analyses of drug-disease intersection proteins. Bioinformatic analyses of drug-disease intersection proteins. **(A)** Gene ontology annotations, including BP, CC, and MF analysis. **(B)** KEGG pathway enrichment analysis of 49 putative targets. KEGG, Kyoto Encyclopedia of Genes and Genomes; BP, biological process; MF, molecular function; CC, cellular component.

To identify the relevant gene functions, the GO annotation, consisting of the BP, CC, and MF categories, was assayed for the 49 DEGs by using R software. As Figure 5A displays, BP was mainly associated with responses to lipopolysaccharides, responses to molecules of bacterial origin, responses to steroid hormones, and cellular responses to cadmium ion. CC was associated with the RNA polymerase II transcription factor complex, nuclear transcription factor complex, transcription factor complex, mitochondrial outer membrane, and organelle outer membrane. MF was associated with nuclear receptor activity, transcription factor activity, nuclear receptor transcription coactivator activity, Bcl-2 homology domain–binding, and death domain–binding. Furthermore, among the signaling pathways enriched by the KEGG analysis, an association was observed between ALI and the tumor necrosis factor signaling pathway, the nucleotide-binding oligomerization domain-like receptor signaling pathway, the Toll-like receptor signaling pathway, apoptosis, and the interleukin-17 signaling pathway (Figure 5B). These data indicate that the protective effects of AR against ALI are closely linked to these signaling pathways. The three highest-scoring genes, namely *MYC*, *MAPK8*, and *CXCL8*, were selected for further analysis (Figure 4C), and the results revealed a high degree of association with apoptosis during the development of ALI.

### Effect of Astragali Radix on Acetaminophen (N-Acetyl-*p*-Aminophenol)-Induced Liver Injury in Mice *in vivo*

**FIGURE 6:**
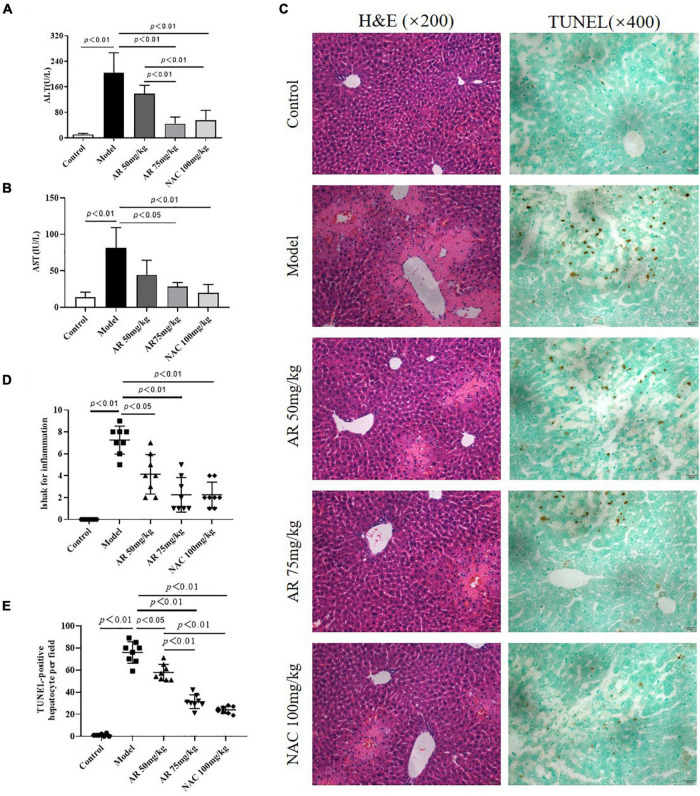
Astragali Radix treatment attenuates APAP-induced liver injury *in vivo*. Mice were administered intragastrically with AR (50, 75 mg/kg body weight) or *N*-acetyl-L-cysteine (NAC, 100 mg/kg body weight) once a day for three consecutive days. Then APAP was dissolved in 5% carboxymethylcellulose sodium in water and given intragastrically with 350 mg/kg body weight as noted in the protocol. Twelve hours after APAP administration, all mice were sacrificed. Serum ALT **(A)** and AST levels **(B)** in AR or NAC treated and untreated mice. **(C)** Representative HE-stained (200×) and TUNEL-stained (magnification 400×) sections of liver tissue from AR or NAC treated and untreated mice 12 h after APAP administration. **(D)** Ishak scores for inflammation from AR treated and untreated mice. **(E)** TUNEL-positive hepatocyte of five high-power fields at ×400 magnification in each tissue specimen.

**FIGURE 7:**
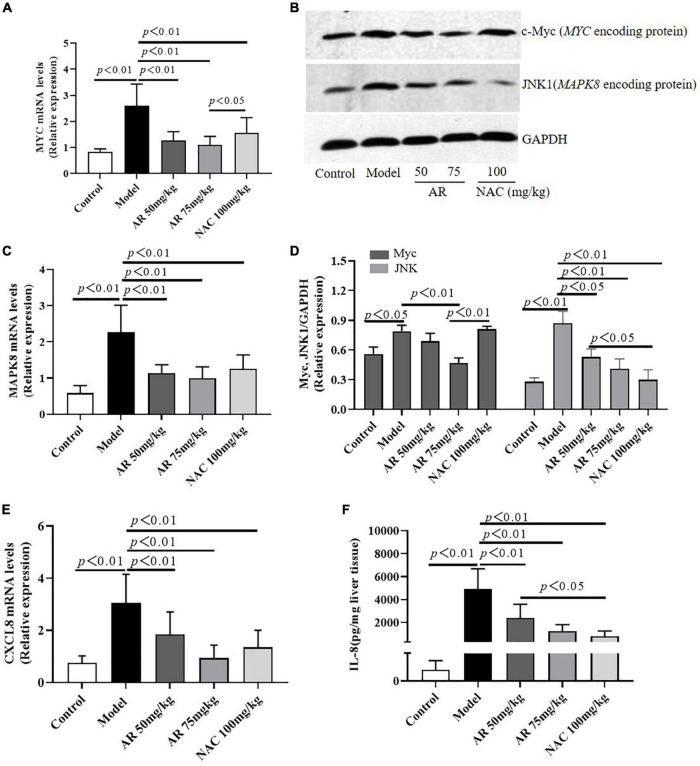
Astragali Radix treatment inhibits the expressions of *MYC* (c-Myc), *MAPK8* (JNK1) and *CXCL8* (IL-8) in APAP-induced liver injury. Mice were administered intragastrically with AR (50, 75 mg/kg body weight) or *N*-acetyl-L-cysteine (NAC, 100 mg/kg body weight) once a day for three consecutive days. Then APAP was dissolved in 5% carboxymethylcellulose sodium in water and given intragastrically with 350 mg/kg body weight as noted in the protocol. Twelve hours after APAP administration, all mice were sacrificed. The mRNA levels of *MYC* **(A)**, *MAPK8* **(C)**, and *CXCL8* **(E)** in liver tissues were assayed by qPCR. **(B)** Western blot analyses for the expressions of c-Myc and JNK1 corresponding to genes *MYC* and *MAPK8* in hepatic tissues and **(D)** graphic presentation of the relative expressions of c-Myc and JNK1. **(E)** The encoding protein IL-8 corresponding to gene *CXCL8* were detected by ELISA. Significantly increased c-Myc, JNK1, and IL-8 expressions were observed in mice of the model group. In contrast, AR treatment attenuated APAP-induced upregulation of c-Myc, JNK1, and IL-8 expressions in dose-dependent. NAC treatment downregulated JNK1 and IL-8 expressions, not affected c-Myc expression.

To evaluate the effect of AR on ALI, serum ALT and AST levels were assayed. After treatment with APAP, the serum ALT and AST levels in the mice increased considerably (Figures 6A,B). In the model mice, hepatocytes in the vicinity of hepatic lesions, particularly those alongside the central vein, exhibited pronounced inflammation and a wide range of necrotic and cytolytic hepatocytes with little mononuclear cell infiltration in the model mice as shown by HE staining (Figure 6C). Likewise, a large number of hepatocyte apoptosis was observed in the model group detected by TUNEL-staining (Figure 6C). The AR groups, especially the AR 75 mg/kg group, exhibited considerable decreases in the ALT and AST levels (Figures 6A,B) and a reduction in liver inflammation and hepatocyte apoptosis (Figures 6C–E) compared with the APAP group. The mice treated with NAC maintained hepatic plate structure, with weaker hepatic lesions alongside the central vein (Figures 6C,D). Fewer TUNEL-positive hepatocytes were observed in the NAC treatment group than in the AR treatment groups (Figures 6C,E). Similarly, AR treatment was also associated with decreased *MYC, MAPK8*, and *CXCL8* mRNA in mouse liver tissues in a dose-dependent manner (Figures 7A,C,E). The encoding proteins c-Myc, JNK1, and IL-8, respectively corresponding to genes *MYC, MAPK8, and CXCL8* were detected by western blotting analysis or ELISA, and the results were consistent with the changes in genes (Figures 7B,D,F). Together, these results suggested that AR attenuated APAP-induced liver injury, partially better than NAC.

### Effects of Astragali Radix Treatment on Acetaminophen (N-Acetyl-*p*-Aminophenol)-Induced Hepatocyte Injury *in vivo*

**FIGURE 8:**
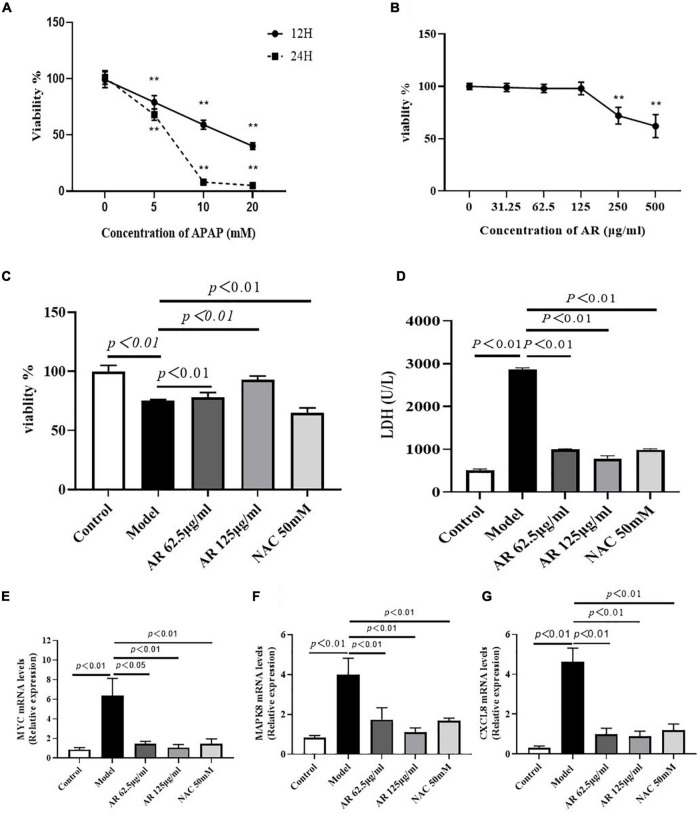
Effect of Astragali Radix (AR) treatment on APAP induced hepatocyte injury and the expression of genes related to hepatocyte apoptosis. **(A)** Viability of L-02 cells at 12 and 24 h incubated with different concentrations of APAP assessed by CCK8 assay. L-02 cells were treated with APAP (20 mM) with or without AR (62.5, 125 μg/ml) and NAC (50 mM) for 12 h. **(B)** Viability of L-02 cells at 12 h incubated with different concentrations of AR observed by CCK8 test. **(C)** Effect of AR on viability of L-02 cells. **(D)** The amount of LDH released into the extracellular medium of AR treatment and untreated L-02 cells. The mRNA levels of *MYC* **(E)**, *MAPK8* **(F)**, and *CXCL8* **(G)** in L-02 cells were assayed.

To verify the effects of AR *in vitro*, hepatocellular injury was induced on the L-02 cells by using APAP. First, the L-02 cells were exposed to 5–20 mM APAP with 2 FC for 12 or 24 h. As Figure 8A displays, the cell viability sharply declined in a dose- and time-dependent manner. APAP substantially inhibited the growth of the L-02 cells, with IC_50_ measuring at approximately 10 mM after 12 h *in vitro*. Second, the cells were cultured with 31.5–500 μg/mL AR for 12 h. After 12 h, the survival rate of the L-02 cells incubated with 31.25–125 μg/mL AR exceeded 95% (Figure 8B), which indicated non-toxicity to the cells. Third, the L-02 cells were exposed to 20 mM APAP for 12 h to induce hepatocellular injury *in vitro*. The cells were then cultured with either 62.5–125 μg/mL AR for treatment or 50 mM NAC for the positive control. After treatment with 62.5 and 125 μg/mL AR, cell viability markedly increased, and LDH content markedly decreased, which were the same as the results observed in the NAC group (Figures 8C,D). These results suggest that AR can protect hepatocytes against APAP *in vitro*. In this study, *MYC, MAPK8*, and *CXCL8* were identified as the potential targets of AR on the basis of network pharmacology. To determine whether *MYC, MAPK8*, and *CXCL8* were involved in the effects of AR on ALI as predicted *in vitro*, mRNA expression was measured after the AR treatment. As illustrated in Figures 8E–G, the expression of *MYC, MAPK8*, and *CXCL8* in the L-02 cells treated with APAP or NAC decreased substantially after the 12 h AR or NAC treatment. These results indicate that AR mitigated the effects of liver injury through the identified target genes and signaling pathways, especially the apoptotic signaling pathway.

### Effects of Astragali Radix on Acetaminophen (*N*-Acetyl-*p*-Aminophenol)-Induced Hepatocyte Apoptosis *in vitro*

**FIGURE 9:**
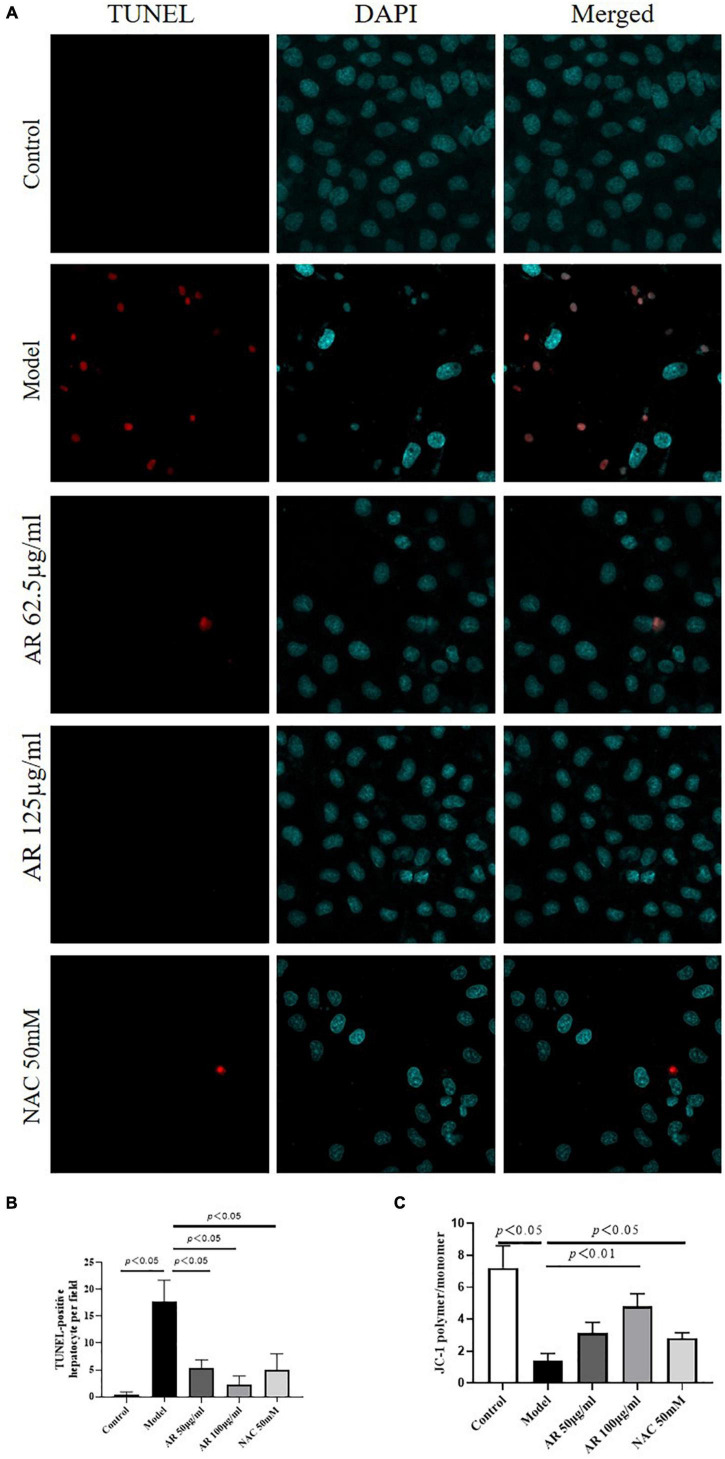
Protective effects of Astragali Radix (AR) on APAP induced hepatocyte injury in L-02 cells. **(A)** The typical images of TUNEL staining (original magnification, 600×; blue: 4’,6-diamidino-2-phenylindole (DAPI); red: TUNEL). **(B)** TUNEL-positive hepatocyte of ten high-power fields at ×600 magnification in each field. **(C)** Semi quantification data for expression of MMP in L-02 cells by examining the fluorescence intensity ratio of JC-1 aggregation/JC-1 monomer.

Because *MYC, MAPK8*, and *CXCL8* levels were highly correlated with cell apoptosis (26–28), then we investigated the effects of AR on apoptosis in the L-02 cells after APAP incubation. As shown in Figures 9A,B, fewer TUNEL-positive hepatocytes were observed in the AR treatment group than in the control model group. High dosages of AR more noticeably improve cell apoptosis than did the NAC treatment *in vitro*. To confirm the protective effects of AR against cell apoptosis, we assayed the expression of MMP in the L-02 cells by examining the fluorescence intensity ratio of JC-1 aggregates to monomers. The semiquantification data on JC-1 revealed the same trend (Figure 9C) as in the AR treatment. These results indicate that AR can inhibit APAP-induced hepatocellular injury by reducing apoptosis *in vitro*.

## Discussion

Drug induced liver injury (DILI) is the main cause of acute liver failure and caused by an excess accumulation of drugs toxic to the metabolism of the human body. This accumulation results in the production of severe oxidative stress, inflammation, and apoptosis, leading to hepatocyte necrosis, which damages the liver. APAP, also known as paracetamol, is a common antipyretic and analgesic drug. Because of its wide range of applications in clinical treatment, APAP is often overused both intentionally and unintentionally, which can lead to severe liver injury, such as DILI and even liver failure. NAC is the most commonly used drug to treat DILI, especially ALI. However, NAC becomes less effective as the disease persists (7). An effective medicine for inhibiting the progression of ALI has yet to be identified and therefore must be developed.

Herbal medicines comprise various active components that increase immunity, boost antioxidation, and inhibit the release of proinflammatory factors through comprehensive multichannel and multitarget regulation. Herbal medicines have remarkable curative effects, such as reducing the effects of toxic substances, relieving clinical symptoms, preventing ALI, and delaying disease progression. TCM can produce synergistic or antagonistic interactions among components through its multicomponent and multitarget principles (29). A network pharmacology approach can elucidate the underlying mechanism of AR in ALI because this approach involves predicting the target profiles and pharmacological mechanisms of active substances in herbal medicines, thereby standardizing and systematizing the interactions among drugs and organisms (30). Similar study has also reported that the most important biotargets for Radix Astragali were caspase 3, MAPK8, MYC (31). We used a network pharmacology approach to investigate the effects of AR on ALI and mechanisms underlying such effects and examined ALI models *in vivo* and *in vitro*.

Astragali Radix, the dried root of *Astragalus membranaceus* (Fisch.) Bge. var. *mongholicus* (Bge.) Hsiao, or *A. membranaceus* (Fisch.) Bge., offers a diverse range of therapeutic effects for liver injury (8, 11). TCM often involves oral administration of medicine in clinical treatment. The oral bioavailability (OB) (32) and the drug-likeness (DL) (33), two ADME-related models, are the main factors affecting the absorption of drugs in the gastrointestinal tract. Therefore, we screened the bioactive components of AR by using two parameters: OB ≥ 30% and DL ≥ 0.18. A total of 181 genes were identified as AR-related genes. To identify the most useful prognostic biomarkers of ALI, we used a bioinformatical method based on the GSE74000 profile dataset and the GeneCards and OMIN databases. By using the VennDiagram package in the R software, we identified a total of 49 DEGs. To understand the relevant gene functions and the possible pathways of the 49 DEGs, GO, and KEGG pathway enrichment was analyzed through R software. The results reveal that the three highest-scoring genes, *MYC, MAPK8*, and *CXCL8*, were markedly enriched in the apoptotic signaling pathway. To ensure the targets had been accurately identified, we investigated the protective effects of AR against ALI *in vivo* and *in vitro*.

Liver injury is associated with two types of hepatic cell death: necrosis and apoptosis (31). Apoptosis plays a vital role in balancing the physiological functions of the organs (34). The serum levels of aminotransferases and the apoptosis of TUNEL-positive hepatocytes increased in the mice with ALI. The histological results show that inflammation and coagulative necrosis were pronounced in the APAP-treated mice. AR treatment reduced the levels of ALT and AST, thereby alleviating liver inflammation, reducing the number of TUNEL-positive hepatocytes and downregulated the expressions *MYC* (c-Myc), *MAPK8* (JNK1), and *CXCL8* (IL-8) in a dose-dependent manner *in vivo*. In the *in vitro* study, we confirmed that the mechanism underlying the protective effect of AR against ALI primarily involved the inhibition of cell apoptosis in hepatocytes accompanied by an increase in hepatocellular viability and a decrease in the release of LDH. We also found that AR significantly inhibited the expressions of *MYC, MAPK8*, and *CXCL8* in the L-02 cells after exposure to APAP. In addition, AR treatment effectively reduced apoptosis in the L-02 cells and accelerated the recovery of the liver *in vitro*. Therefore, preventing the activation of the apoptotic pathway is a vital component of the mechanism through which AR inhibits the progression of ALI.

## Conclusion

In conclusion, the pharmacological mechanism by which AR alleviated ALI was investigated with combination of network pharmacology prediction and experimental validation. We demonstrated that AR may decrease hepatocyte necrosis and apoptosis associated with downregulating the expressions of *MYC* (c-Myc), *MAPK8* (JNK1), and *CXCL8* (IL-8). The potential therapeutic effects of AR on ALI may benefit from the further studies on clinical trials of ALI patients with AR treatment.

## Data Availability Statement

The accession numbers for the data generated/analyzed in this study can be found in the manuscript or Supplementary Material.

## Ethics Statement

The animal study was reviewed and approved by Shuguang Hospital, affiliated with Shanghai University of Traditional Chinese Medicine. The protocols were reviewed and approved by the Ethics Committee of the institution. Written informed consent was obtained from the owners for the participation of their animals in this study.

## Author Contributions

YT and CL conceived and designed the project. YP, GZ, YM, GC, and KH conducted the experiments. YP and YT analyzed the data and wrote the manuscript. CL revised the manuscript. YT made the final critical revision to the manuscript. All authors contributed to the article and approved the submitted version.

## Conflict of Interest

The authors declare that the research was conducted in the absence of any commercial or financial relationships that could be construed as a potential conflict of interest.

## Publisher’s Note

All claims expressed in this article are solely those of the authors and do not necessarily represent those of their affiliated organizations, or those of the publisher, the editors and the reviewers. Any product that may be evaluated in this article, or claim that may be made by its manufacturer, is not guaranteed or endorsed by the publisher.

## Footnotes

1
http://tcmspw.com/tcmsp.php

2
http://bionet.ncpsb.org.cn/batman-tcm/

3
https://pubchem.ncbi.nlm.nih.gov

4
www.drugbank.ca

5
https://www.ncbi.nlm.nih.gov/geo

6
http://www.genecards.org

7
http://omim.org/

8
https://string-db.org

9
http://www.geneontology.org

10
http://www.genome.jp/kegg

## Abbreviations

ADME: absorption distribution, metabolism and excretion
ALI: acetaminophen-induced liver injury
ALT: alanine aminotransferase
APAP: acetaminophen (*N*-acetyl-p-aminophenol)
AR: astragali radix
AST: aspartate aminotransferase
BATMAN-TCM: bioinformatics analysis tool for molecular mechanism of traditional Chinese medicine
BP: biological process
CC: cellular component
DEGs: expressed genes
DL: drug-likeness
ELISA: enzyme-linked immunosorbent assay
GO: gene ontology
GSH: glutathione
KEGG: Kyoto Encyclopedia of Genes and Genomes
LDH: lactate dehydrogenase
MF: molecular function
NAC: *N*-acetylcysteine
NAPQI: *N*-acetyl -p-benzoquinone imine
OB: oral bioavailability
OMIM: Online Mendelian Inheritance in Man
PBS: phosphate-buffered saline
PPI: protein–protein interaction
TCM: traditional Chinese medicine
TCMID: traditional Chinese medicine integrated database
TCMSP: traditional Chinese medicine systems pharmacology
TUNEL: terminal deoxyuridine triphosphate nick-end labeling.
