# Supplementary material for: Network Pharmacology–Based Prediction and Pharmacological Validation of Effects of Astragali Radix on Acetaminophen-Induced Liver Injury
Source: Front Med (Lausanne). 2022 Jul 4;9:697644. doi: 10.3389/fmed.2022.697644 (PMC9289209; doi:10.3389/fmed.2022.697644)
Supplement: Supplementary file 1 [file Table_1.DOCX]

| Molecule name | Target name | Source |
| --- | --- | --- |
| Jaranol | Dipeptidyl peptidase IV | DrugBank |
| Jaranol | Calmodulin | DrugBank |
| Jaranol | Prostaglandin G/H synthase 1 | DrugBank |
| Jaranol | Androgen receptor | DrugBank |
| Jaranol | Sodium channel protein type 5 subunit alpha | DrugBank |
| Jaranol | Prostaglandin G/H synthase 2 | DrugBank |
| Jaranol | Estrogen receptor beta | DrugBank |
| Jaranol | Nitric oxide synthase, inducible | DrugBank |
| Jaranol | Heat shock protein HSP 90 | DrugBank |
| Jaranol | Cell division protein kinase 2 | DrugBank |
| Jaranol | Serine/threonine-protein kinase Chk1 | DrugBank |
| Jaranol | Trypsin-1 | DrugBank |
| Jaranol | Nuclear receptor coactivator 2 | DrugBank |
| Mairin | Progesterone receptor | DrugBank |
| (3S,8S,9S,10R,13R,14S,17R)-10,13-dimethyl-17-[(2R,5S)-5-propan-2-yloctan-2-yl]-2,3,4,7,8,9,11,12,14,15,16,17-dodecahydro-1H-cyclopenta[a]phenanthren-3-ol | Progesterone receptor | DrugBank |
| hederagenin | Progesterone receptor | DrugBank |
| hederagenin | Muscarinic acetylcholine receptor M3 | DrugBank |
| hederagenin | Muscarinic acetylcholine receptor M1 | DrugBank |
| hederagenin | Gamma-aminobutyric-acid receptor alpha-2 subunit | DrugBank |
| hederagenin | Gamma-aminobutyric-acid receptor alpha-3 subunit | DrugBank |
| hederagenin | Muscarinic acetylcholine receptor M2 | DrugBank |
| hederagenin | Alpha-1B adrenergic receptor | DrugBank |
| hederagenin | Gamma-aminobutyric acid receptor subunit alpha-1 | DrugBank |
| hederagenin | Glutamate receptor 2 | DrugBank |
| hederagenin | Gamma-aminobutyric-acid receptor subunit alpha-6 | DrugBank |
| hederagenin | Gamma-aminobutyric-acid receptor alpha-5 subunit | DrugBank |
| hederagenin | Nuclear receptor coactivator 2 | DrugBank |
| hederagenin | Alcohol dehydrogenase 1B | DrugBank |
| hederagenin | Alcohol dehydrogenase 1C | DrugBank |
| hederagenin | Lysozyme | DrugBank |
| hederagenin | Nicotinate-nucleotide--dimethylbenzimidazole phosphoribosyltransferase | DrugBank |
| hederagenin | Prostaglandin G/H synthase 1 | DrugBank |
| hederagenin | Sodium channel protein type 5 subunit alpha | DrugBank |
| hederagenin | Prostaglandin G/H synthase 2 | DrugBank |
| hederagenin | Retinoic acid receptor RXR-alpha | DrugBank |
| hederagenin | CGMP-inhibited 3',5'-cyclic phosphodiesterase A | DrugBank |
| hederagenin | Sodium-dependent noradrenaline transporter | DrugBank |
| hederagenin | Cytochrome P450-cam | DrugBank |
| hederagenin | Ig gamma-1 chain C region | DrugBank |
| 3,9-di-O-methylnissolin | 5-hydroxytryptamine receptor 3A | DrugBank |
| 3,9-di-O-methylnissolin | Calmodulin | DrugBank |
| 3,9-di-O-methylnissolin | Prostaglandin G/H synthase 1 | DrugBank |
| 3,9-di-O-methylnissolin | Muscarinic acetylcholine receptor M3 | DrugBank |
| 3,9-di-O-methylnissolin | Thrombin | DrugBank |
| 3,9-di-O-methylnissolin | Muscarinic acetylcholine receptor M1 | DrugBank |
| 3,9-di-O-methylnissolin | Estrogen receptor | DrugBank |
| 3,9-di-O-methylnissolin | Beta-1 adrenergic receptor | DrugBank |
| 3,9-di-O-methylnissolin | Sodium channel protein type 5 subunit alpha | DrugBank |
| 3,9-di-O-methylnissolin | Prostaglandin G/H synthase 2 | DrugBank |
| 3,9-di-O-methylnissolin | Nitric-oxide synthase, endothelial | DrugBank |
| 3,9-di-O-methylnissolin | Nitric oxide synthase, inducible | DrugBank |
| 3,9-di-O-methylnissolin | Alpha-2C adrenergic receptor | DrugBank |
| 3,9-di-O-methylnissolin | Retinoic acid receptor RXR-alpha | DrugBank |
| 3,9-di-O-methylnissolin | Acetylcholinesterase | DrugBank |
| 3,9-di-O-methylnissolin | CGMP-inhibited 3',5'-cyclic phosphodiesterase A | DrugBank |
| 3,9-di-O-methylnissolin | Alpha-1B adrenergic receptor | DrugBank |
| 3,9-di-O-methylnissolin | Beta-2 adrenergic receptor | DrugBank |
| 3,9-di-O-methylnissolin | Alpha-1D adrenergic receptor | DrugBank |
| 3,9-di-O-methylnissolin | Mu-type opioid receptor | DrugBank |
| 3,9-di-O-methylnissolin | Gamma-aminobutyric acid receptor subunit alpha-1 | DrugBank |
| 3,9-di-O-methylnissolin | Trypsin-1 | DrugBank |
| 3,9-di-O-methylnissolin | Nuclear receptor coactivator 2 | DrugBank |
| isorhamnetin | Aldose reductase | DrugBank |
| isorhamnetin | Nuclear receptor coactivator 1 | DrugBank |
| isorhamnetin | Coagulation factor VII | DrugBank |
| isorhamnetin | Thrombin | DrugBank |
| isorhamnetin | Nitric-oxide synthase, endothelial | DrugBank |
| isorhamnetin | Acetylcholinesterase | DrugBank |
| isorhamnetin | Gamma-aminobutyric acid receptor subunit alpha-1 | DrugBank |
| isorhamnetin | Prostaglandin G/H synthase 1 | DrugBank |
| isorhamnetin | Glutamate receptor 2 | DrugBank |
| isorhamnetin | Cytochrome P450-cam | DrugBank |
| isorhamnetin | Transcription factor p65 | N/A |
| isorhamnetin | Xanthine dehydrogenase/oxidase | DrugBank |
| isorhamnetin | Neutrophil cytosol factor 1 | N/A |
| isorhamnetin | Oxidized low-density lipoprotein receptor 1 | N/A |
| isorhamnetin | Nitric oxide synthase, inducible | DrugBank |
| isorhamnetin | Estrogen receptor | DrugBank |
| isorhamnetin | Androgen receptor | DrugBank |
| isorhamnetin | Peroxisome proliferator activated receptor gamma | DrugBank |
| isorhamnetin | Prostaglandin G/H synthase 2 | DrugBank |
| isorhamnetin | mRNA of Protein-tyrosine phosphatase, non-receptor type 1 | DrugBank |
| isorhamnetin | Estrogen receptor beta | DrugBank |
| isorhamnetin | Dipeptidyl peptidase IV | DrugBank |
| isorhamnetin | Mitogen-activated protein kinase 14 | DrugBank |
| isorhamnetin | Glycogen synthase kinase-3 beta | DrugBank |
| isorhamnetin | Heat shock protein HSP 90 | DrugBank |
| isorhamnetin | Cell division protein kinase 2 | DrugBank |
| isorhamnetin | Phosphatidylinositol-4,5-bisphosphate 3-kinase catalytic subunit, gamma isoform | DrugBank |
| isorhamnetin | mRNA of PKA Catalytic Subunit C-alpha | DrugBank |
| isorhamnetin | Trypsin-1 | DrugBank |
| isorhamnetin | Proto-oncogene serine/threonine-protein kinase Pim-1 | DrugBank |
| isorhamnetin | Cyclin-A2 | DrugBank |
| isorhamnetin | Nuclear receptor coactivator 2 | DrugBank |
| isorhamnetin | Calmodulin | DrugBank |
| isorhamnetin | Glycogen phosphorylase, muscle form | DrugBank |
| isorhamnetin | Peroxisome proliferator activated receptor delta | DrugBank |
| isorhamnetin | Serine/threonine-protein kinase Chk1 | DrugBank |
| isorhamnetin | Amine oxidase [flavin-containing] B | DrugBank |
| isorhamnetin | Mitogen-activated protein kinase 14 | DrugBank |
| isorhamnetin | Glycogen synthase kinase-3 beta | DrugBank |
| isorhamnetin | Heat shock protein HSP 90 | DrugBank |
| isorhamnetin | Cell division protein kinase 2 | DrugBank |
| isorhamnetin | Phosphatidylinositol-4,5-bisphosphate 3-kinase catalytic subunit, gamma isoform | DrugBank |
| isorhamnetin | mRNA of PKA Catalytic Subunit C-alpha | DrugBank |
| isorhamnetin | Trypsin-1 | DrugBank |
| isorhamnetin | Proto-oncogene serine/threonine-protein kinase Pim-1 | DrugBank |
| isorhamnetin | Cyclin-A2 | DrugBank |
| isorhamnetin | Nuclear receptor coactivator 2 | DrugBank |
| isorhamnetin | Calmodulin | DrugBank |
| isorhamnetin | Glycogen phosphorylase, muscle form | DrugBank |
| isorhamnetin | Peroxisome proliferator activated receptor delta | DrugBank |
| isorhamnetin | Serine/threonine-protein kinase Chk1 | DrugBank |
| isorhamnetin | Amine oxidase [flavin-containing] B | DrugBank |
| 7-O-methylisomucronulatol | Alpha-1A adrenergic receptor | DrugBank |
| 7-O-methylisomucronulatol | Nitric oxide synthase, inducible | DrugBank |
| 7-O-methylisomucronulatol | Dopamine D1 receptor | DrugBank |
| 7-O-methylisomucronulatol | Muscarinic acetylcholine receptor M3 | DrugBank |
| 7-O-methylisomucronulatol | Thrombin | DrugBank |
| 7-O-methylisomucronulatol | Potassium voltage-gated channel subfamily H member 2 | DrugBank |
| 7-O-methylisomucronulatol | Muscarinic acetylcholine receptor M1 | DrugBank |
| 7-O-methylisomucronulatol | Estrogen receptor | DrugBank |
| 7-O-methylisomucronulatol | Androgen receptor | DrugBank |
| 7-O-methylisomucronulatol | Beta-1 adrenergic receptor | DrugBank |
| 7-O-methylisomucronulatol | Sodium channel protein type 5 subunit alpha | DrugBank |
| 7-O-methylisomucronulatol | Peroxisome proliferator activated receptor gamma | DrugBank |
| 7-O-methylisomucronulatol | Coagulation factor Xa | DrugBank |
| 7-O-methylisomucronulatol | Muscarinic acetylcholine receptor M5 | DrugBank |
| 7-O-methylisomucronulatol | Prostaglandin G/H synthase 2 | DrugBank |
| 7-O-methylisomucronulatol | Nitric-oxide synthase, endothelial | DrugBank |
| 7-O-methylisomucronulatol | Alpha-2C adrenergic receptor | DrugBank |
| 7-O-methylisomucronulatol | Muscarinic acetylcholine receptor M4 | DrugBank |
| 7-O-methylisomucronulatol | Retinoic acid receptor RXR-alpha | DrugBank |
| 7-O-methylisomucronulatol | Delta-type opioid receptor | DrugBank |
| 7-O-methylisomucronulatol | CGMP-inhibited 3',5'-cyclic phosphodiesterase A | DrugBank |
| 7-O-methylisomucronulatol | 5-hydroxytryptamine 2A receptor | DrugBank |
| 7-O-methylisomucronulatol | Prostaglandin G/H synthase 1 | DrugBank |
| 7-O-methylisomucronulatol | Muscarinic acetylcholine receptor M2 | DrugBank |
| 7-O-methylisomucronulatol | Alpha-1B adrenergic receptor | DrugBank |
| 7-O-methylisomucronulatol | Sodium-dependent dopamine transporter | DrugBank |
| 7-O-methylisomucronulatol | Beta-2 adrenergic receptor | DrugBank |
| 7-O-methylisomucronulatol | Alpha-1D adrenergic receptor | DrugBank |
| 7-O-methylisomucronulatol | Sodium-dependent serotonin transporter | DrugBank |
| 7-O-methylisomucronulatol | Estrogen receptor beta | DrugBank |
| 7-O-methylisomucronulatol | Gamma-aminobutyric acid receptor subunit alpha-1 | DrugBank |
| 7-O-methylisomucronulatol | Dipeptidyl peptidase IV | DrugBank |
| 7-O-methylisomucronulatol | Mitogen-activated protein kinase 14 | DrugBank |
| 7-O-methylisomucronulatol | Glycogen synthase kinase-3 beta | DrugBank |
| 7-O-methylisomucronulatol | Heat shock protein HSP 90 | DrugBank |
| 7-O-methylisomucronulatol | Cell division protein kinase 2 | DrugBank |
| 7-O-methylisomucronulatol | Serine/threonine-protein kinase Chk1 | DrugBank |
| 7-O-methylisomucronulatol | mRNA of PKA Catalytic Subunit C-alpha | DrugBank |
| 7-O-methylisomucronulatol | Retinoic acid receptor RXR-beta | DrugBank |
| 7-O-methylisomucronulatol | Trypsin-1 | DrugBank |
| 7-O-methylisomucronulatol | Proto-oncogene serine/threonine-protein kinase Pim-1 | DrugBank |
| 7-O-methylisomucronulatol | Cyclin-A2 | DrugBank |
| 7-O-methylisomucronulatol | Nuclear receptor coactivator 2 | DrugBank |
| 7-O-methylisomucronulatol | Calcium-activated potassium channel subunit alpha 1 | DrugBank |
| 7-O-methylisomucronulatol | Calmodulin | DrugBank |
| (6aR,11aR)-9,10-dimethoxy-6a,11a-dihydro-6H-benzofurano[3,2-c]chromen-3-ol | Retinoic acid receptor RXR-alpha | DrugBank |
| (6aR,11aR)-9,10-dimethoxy-6a,11a-dihydro-6H-benzofurano[3,2-c]chromen-3-ol | Muscarinic acetylcholine receptor M4 | DrugBank |
| (6aR,11aR)-9,10-dimethoxy-6a,11a-dihydro-6H-benzofurano[3,2-c]chromen-3-ol | Calmodulin | DrugBank |
| (6aR,11aR)-9,10-dimethoxy-6a,11a-dihydro-6H-benzofurano[3,2-c]chromen-3-ol | Nitric oxide synthase, inducible | DrugBank |
| (6aR,11aR)-9,10-dimethoxy-6a,11a-dihydro-6H-benzofurano[3,2-c]chromen-3-ol | Prostaglandin G/H synthase 1 | DrugBank |
| (6aR,11aR)-9,10-dimethoxy-6a,11a-dihydro-6H-benzofurano[3,2-c]chromen-3-ol | Muscarinic acetylcholine receptor M3 | DrugBank |
| (6aR,11aR)-9,10-dimethoxy-6a,11a-dihydro-6H-benzofurano[3,2-c]chromen-3-ol | Thrombin | DrugBank |
| (6aR,11aR)-9,10-dimethoxy-6a,11a-dihydro-6H-benzofurano[3,2-c]chromen-3-ol | Muscarinic acetylcholine receptor M1 | DrugBank |
| (6aR,11aR)-9,10-dimethoxy-6a,11a-dihydro-6H-benzofurano[3,2-c]chromen-3-ol | Estrogen receptor | DrugBank |
| (6aR,11aR)-9,10-dimethoxy-6a,11a-dihydro-6H-benzofurano[3,2-c]chromen-3-ol | Sodium channel protein type 5 subunit alpha | DrugBank |
| (6aR,11aR)-9,10-dimethoxy-6a,11a-dihydro-6H-benzofurano[3,2-c]chromen-3-ol | Prostaglandin G/H synthase 2 | DrugBank |
| (6aR,11aR)-9,10-dimethoxy-6a,11a-dihydro-6H-benzofurano[3,2-c]chromen-3-ol | 5-hydroxytryptamine receptor 3A | DrugBank |
| (6aR,11aR)-9,10-dimethoxy-6a,11a-dihydro-6H-benzofurano[3,2-c]chromen-3-ol | Nuclear receptor coactivator 1 | DrugBank |
| (6aR,11aR)-9,10-dimethoxy-6a,11a-dihydro-6H-benzofurano[3,2-c]chromen-3-ol | Acetylcholinesterase | DrugBank |
| (6aR,11aR)-9,10-dimethoxy-6a,11a-dihydro-6H-benzofurano[3,2-c]chromen-3-ol | Alpha-1B adrenergic receptor | DrugBank |
| (6aR,11aR)-9,10-dimethoxy-6a,11a-dihydro-6H-benzofurano[3,2-c]chromen-3-ol | Beta-2 adrenergic receptor | DrugBank |
| (6aR,11aR)-9,10-dimethoxy-6a,11a-dihydro-6H-benzofurano[3,2-c]chromen-3-ol | Alpha-1D adrenergic receptor | DrugBank |
| (6aR,11aR)-9,10-dimethoxy-6a,11a-dihydro-6H-benzofurano[3,2-c]chromen-3-ol | Gamma-aminobutyric acid receptor subunit alpha-1 | DrugBank |
| (6aR,11aR)-9,10-dimethoxy-6a,11a-dihydro-6H-benzofurano[3,2-c]chromen-3-ol | Heat shock protein HSP 90 | DrugBank |
| (6aR,11aR)-9,10-dimethoxy-6a,11a-dihydro-6H-benzofurano[3,2-c]chromen-3-ol | Neuronal acetylcholine receptor protein, alpha-7 chain | DrugBank |
| (6aR,11aR)-9,10-dimethoxy-6a,11a-dihydro-6H-benzofurano[3,2-c]chromen-3-ol | Trypsin-1 | DrugBank |
| (6aR,11aR)-9,10-dimethoxy-6a,11a-dihydro-6H-benzofurano[3,2-c]chromen-3-ol | Nuclear receptor coactivator 2 | DrugBank |
| 9,10-dimethoxypterocarpan-3-O-β-D-glucoside | DNA topoisomerase II | DrugBank |
| 9,10-dimethoxypterocarpan-3-O-β-D-glucoside | Nuclear receptor coactivator 2 | DrugBank |
| 9,10-dimethoxypterocarpan-3-O-β-D-glucoside | Prostaglandin G/H synthase 2 | DrugBank |
| Bifendate | Prostaglandin G/H synthase 2 | DrugBank |
| Bifendate | Hepatocyte growth factor receptor | DrugBank |
| Bifendate | Heat shock protein HSP 90 | DrugBank |
| Bifendate | Calcium-activated potassium channel subunit alpha 1 | DrugBank |
| Bifendate | Prostaglandin G/H synthase 1 | DrugBank |
| Bifendate | DNA topoisomerase II | DrugBank |
| Bifendate | Vascular endothelial growth factor receptor 2 | DrugBank |
| formononetin | Nitric oxide synthase, inducible | DrugBank |
| formononetin | Prostaglandin G/H synthase 1 | DrugBank |
| formononetin | Muscarinic acetylcholine receptor M1 | DrugBank |
| formononetin | Estrogen receptor | DrugBank |
| formononetin | Androgen receptor | DrugBank |
| formononetin | Peroxisome proliferator activated receptor gamma | DrugBank |
| formononetin | Prostaglandin G/H synthase 2 | DrugBank |
| formononetin | Retinoic acid receptor RXR-alpha | DrugBank |
| formononetin | CGMP-inhibited 3',5'-cyclic phosphodiesterase A | DrugBank |
| formononetin | Alpha-1A adrenergic receptor | DrugBank |
| formononetin | Sodium-dependent dopamine transporter | DrugBank |
| formononetin | Beta-2 adrenergic receptor | DrugBank |
| formononetin | Sodium-dependent serotonin transporter | DrugBank |
| formononetin | Estrogen receptor beta | DrugBank |
| formononetin | Dipeptidyl peptidase IV | DrugBank |
| formononetin | Mitogen-activated protein kinase 14 | DrugBank |
| formononetin | 3 beta-hydroxysteroid dehydrogenase/Delta 5-->4-isomerase type 1 | N/A |
| formononetin | Heat shock protein HSP 90 | DrugBank |
| formononetin | Cell division protein kinase 2 | DrugBank |
| formononetin | Amine oxidase [flavin-containing] B | DrugBank |
| formononetin | Serine/threonine-protein kinase Chk1 | DrugBank |
| formononetin | mRNA of PKA Catalytic Subunit C-alpha | DrugBank |
| formononetin | Trypsin-1 | DrugBank |
| formononetin | Proto-oncogene serine/threonine-protein kinase Pim-1 | DrugBank |
| formononetin | Cyclin-A2 | DrugBank |
| formononetin | Calmodulin | DrugBank |
| formononetin | cAMP-dependent protein kinase inhibitor alpha | DrugBank |
| formononetin | Thrombin | DrugBank |
| formononetin | Nitric-oxide synthase, endothelial | DrugBank |
| formononetin | Acetylcholinesterase | DrugBank |
| formononetin | Beta-lactamase | DrugBank |
| formononetin | Transcription factor AP-1 | DrugBank |
| formononetin | Peroxisome proliferator-activated receptor gamma | N/A |
| formononetin | Interleukin-4 | N/A |
| formononetin | NAD-dependent deacetylase sirtuin-1 | N/A |
| formononetin | ATP synthase subunit beta, mitochondrial | DrugBank |
| formononetin | NADH-ubiquinone oxidoreductase chain 6 | DrugBank |
| formononetin | 3 beta-hydroxysteroid dehydrogenase/Delta 5-->4-isomerase type 2 | N/A |
| formononetin | Glycogen synthase kinase-3 beta | DrugBank |
| Calycosin | Glycogen synthase kinase-3 beta | DrugBank |
| Calycosin | Nitric oxide synthase, inducible | DrugBank |
| Calycosin | Estrogen receptor | DrugBank |
| Calycosin | Androgen receptor | DrugBank |
| Calycosin | Peroxisome proliferator activated receptor gamma | DrugBank |
| Calycosin | Prostaglandin G/H synthase 2 | DrugBank |
| Calycosin | Retinoic acid receptor RXR-alpha | DrugBank |
| Calycosin | CGMP-inhibited 3',5'-cyclic phosphodiesterase A | DrugBank |
| Calycosin | Estrogen receptor beta | DrugBank |
| Calycosin | Dipeptidyl peptidase IV | DrugBank |
| Calycosin | Mitogen-activated protein kinase 14 | DrugBank |
| Calycosin | Prostaglandin G/H synthase 1 | DrugBank |
| Calycosin | Heat shock protein HSP 90 | DrugBank |
| Calycosin | Cell division protein kinase 2 | DrugBank |
| Calycosin | Serine/threonine-protein kinase Chk1 | DrugBank |
| Calycosin | mRNA of PKA Catalytic Subunit C-alpha | DrugBank |
| Calycosin | Trypsin-1 | DrugBank |
| Calycosin | Proto-oncogene serine/threonine-protein kinase Pim-1 | DrugBank |
| Calycosin | Cyclin-A2 | DrugBank |
| Calycosin | Nuclear receptor coactivator 2 | DrugBank |
| Calycosin | Calmodulin | DrugBank |
| Calycosin | Beta-2 adrenergic receptor | DrugBank |
| FA | Glycogen synthase kinase-3 beta | DrugBank |
| FA | Cell division protein kinase 2 | DrugBank |
| FA | Thrombin | DrugBank |
| kaempferol | Peroxisome proliferator activated receptor gamma | DrugBank |
| kaempferol | Prostaglandin G/H synthase 2 | DrugBank |
| kaempferol | Heat shock protein HSP 90 | DrugBank |
| kaempferol | Phosphatidylinositol-4,5-bisphosphate 3-kinase catalytic subunit, gamma isoform | DrugBank |
| kaempferol | mRNA of PKA Catalytic Subunit C-alpha | DrugBank |
| kaempferol | Nuclear receptor coactivator 2 | DrugBank |
| kaempferol | Dipeptidyl peptidase IV | DrugBank |
| kaempferol | Trypsin-1 | DrugBank |
| kaempferol | Progesterone receptor | DrugBank |
| kaempferol | Thrombin | DrugBank |
| kaempferol | Muscarinic acetylcholine receptor M1 | DrugBank |
| kaempferol | Nitric-oxide synthase, endothelial | DrugBank |
| kaempferol | Gamma-aminobutyric-acid receptor alpha-2 subunit | DrugBank |
| kaempferol | Acetylcholinesterase | DrugBank |
| kaempferol | Sodium-dependent noradrenaline transporter | DrugBank |
| kaempferol | Muscarinic acetylcholine receptor M2 | DrugBank |
| kaempferol | Alpha-1B adrenergic receptor | DrugBank |
| kaempferol | Gamma-aminobutyric acid receptor subunit alpha-1 | DrugBank |
| kaempferol | DNA topoisomerase II | DrugBank |
| kaempferol | Coagulation factor VII | DrugBank |
| kaempferol | Calmodulin | DrugBank |
| kaempferol | Transcription factor p65 | N/A |
| kaempferol | Inhibitor of nuclear factor kappa-B kinase subunit beta | N/A |
| kaempferol | RAC-alpha serine/threonine-protein kinase | N/A |
| kaempferol | Apoptosis regulator Bcl-2 | DrugBank |
| kaempferol | Apoptosis regulator BAX | N/A |
| kaempferol | Tumor necrosis factor | DrugBank |
| kaempferol | Transcription factor AP-1 | DrugBank |
| kaempferol | Activator of 90 kDa heat shock protein ATPase homolog 1 | N/A |
| kaempferol | Caspase-3 | N/A |
| kaempferol | Prostaglandin G/H synthase 1 | DrugBank |
| kaempferol | Xanthine dehydrogenase/oxidase | DrugBank |
| kaempferol | Interstitial collagenase | DrugBank |
| kaempferol | Signal transducer and activator of transcription 1-alpha/beta | N/A |
| kaempferol | Cell division control protein 2 homolog | DrugBank |
| kaempferol | Peroxisome proliferator-activated receptor gamma | N/A |
| kaempferol | Heme oxygenase 1 | DrugBank |
| kaempferol | Cytochrome P450 3A4 | DrugBank |
| kaempferol | Cytochrome P450 1A2 | DrugBank |
| kaempferol | Cytochrome P450 1A1 | N/A |
| kaempferol | Intercellular adhesion molecule 1 | N/A |
| kaempferol | E-selectin | DrugBank |
| kaempferol | Vascular cell adhesion protein 1 | DrugBank |
| kaempferol | Nuclear receptor subfamily 1 group I member 2 | N/A |
| kaempferol | Cytochrome P450 1B1 | N/A |
| kaempferol | Arachidonate 5-lipoxygenase | DrugBank |
| kaempferol | Hyaluronan synthase 2 | N/A |
| kaempferol | Glutathione S-transferase P | DrugBank |
| kaempferol | Aryl hydrocarbon receptor | DrugBank |
| kaempferol | 26S proteasome non-ATPase regulatory subunit 3 | N/A |
| kaempferol | Solute carrier family 2, facilitated glucose transporter member 4 | N/A |
| kaempferol | Nuclear receptor subfamily 1 group I member 3 | N/A |
| kaempferol | Insulin receptor | DrugBank |
| kaempferol | Type I iodothyronine deiodinase | N/A |
| kaempferol | Serine/threonine-protein phosphatase 2B catalytic subunit alpha isoform | DrugBank |
| kaempferol | Peroxidase C1A | N/A |
| kaempferol | Glutathione S-transferase Mu 1 | DrugBank |
| kaempferol | Glutathione S-transferase Mu 2 | DrugBank |
| kaempferol | Aldo-keto reductase family 1 member C3 | DrugBank |
| kaempferol | Antileukoproteinase | N/A |
| kaempferol | Nitric oxide synthase, inducible | DrugBank |
| kaempferol | Androgen receptor | DrugBank |
| kaempferol | Mitogen-activated protein kinase 8 | DrugBank |
| isomucronulatol-7,2'-di-O-glucosiole | DNA topoisomerase II | DrugBank |
| 1,7-Dihydroxy-3,9-dimethoxy pterocarpene | Prostaglandin G/H synthase 2 | DrugBank |
| 1,7-Dihydroxy-3,9-dimethoxy pterocarpene | Heat shock protein HSP 90 | DrugBank |
| 1,7-Dihydroxy-3,9-dimethoxy pterocarpene | Trypsin-1 | DrugBank |
| 1,7-Dihydroxy-3,9-dimethoxy pterocarpene | Retinoic acid receptor RXR-alpha | DrugBank |
| quercetin | Prostaglandin G/H synthase 1 | DrugBank |
| quercetin | Androgen receptor | DrugBank |
| quercetin | Peroxisome proliferator activated receptor gamma | DrugBank |
| quercetin | Prostaglandin G/H synthase 2 | DrugBank |
| quercetin | Heat shock protein HSP 90 | DrugBank |
| quercetin | Phosphatidylinositol-4,5-bisphosphate 3-kinase catalytic subunit, gamma isoform | DrugBank |
| quercetin | Nuclear receptor coactivator 2 | DrugBank |
| quercetin | Dipeptidyl peptidase IV | DrugBank |
| quercetin | Aldose reductase | DrugBank |
| quercetin | Trypsin-1 | DrugBank |
| quercetin | DNA topoisomerase II | DrugBank |
| quercetin | Thrombin | DrugBank |
| quercetin | Potassium voltage-gated channel subfamily H member 2 | DrugBank |
| quercetin | Sodium channel protein type 5 subunit alpha | DrugBank |
| quercetin | Coagulation factor Xa | DrugBank |
| quercetin | Beta-2 adrenergic receptor | DrugBank |
| quercetin | Stromelysin-1 | DrugBank |
| quercetin | mRNA of PKA Catalytic Subunit C-alpha | DrugBank |
| quercetin | Coagulation factor VII | DrugBank |
| quercetin | Nitric-oxide synthase, endothelial | DrugBank |
| quercetin | Retinoic acid receptor RXR-alpha | DrugBank |
| quercetin | Acetylcholinesterase | DrugBank |
| quercetin | Gamma-aminobutyric acid receptor subunit alpha-1 | DrugBank |
| quercetin | Amine oxidase [flavin-containing] B | DrugBank |
| quercetin | Transcription factor p65 | N/A |
| quercetin | Epidermal growth factor receptor | DrugBank |
| quercetin | RAC-alpha serine/threonine-protein kinase | N/A |
| quercetin | Vascular endothelial growth factor A | DrugBank |
| quercetin | G1/S-specific cyclin-D1 | N/A |
| quercetin | Apoptosis regulator Bcl-2 | DrugBank |
| quercetin | Bcl-2-like protein 1 | N/A |
| quercetin | Proto-oncogene c-Fos | N/A |
| quercetin | Cyclin-dependent kinase inhibitor 1 | N/A |
| quercetin | Eukaryotic translation initiation factor 6 | N/A |
| quercetin | Apoptosis regulator BAX | N/A |
| quercetin | Caspase-9 | N/A |
| quercetin | Urokinase-type plasminogen activator | DrugBank |
| quercetin | 72 kDa type IV collagenase | DrugBank |
| quercetin | Matrix metalloproteinase-9 | N/A |
| quercetin | Mitogen-activated protein kinase 1 | DrugBank |
| quercetin | Interleukin-10 | N/A |
| quercetin | Pro-epidermal growth factor | DrugBank |
| quercetin | Retinoblastoma-associated protein | DrugBank |
| quercetin | Tumor necrosis factor | DrugBank |
| quercetin | Transcription factor AP-1 | DrugBank |
| quercetin | Interleukin-6 | DrugBank |
| quercetin | Cyclin-dependent kinase inhibitor 2A, isoforms 1/2/3 | N/A |
| quercetin | Activator of 90 kDa heat shock protein ATPase homolog 1 | N/A |
| quercetin | Caspase-3 | N/A |
| quercetin | Cellular tumor antigen p53 | DrugBank |
| quercetin | ETS domain-containing protein Elk-1 | N/A |
| quercetin | NF-kappa-B inhibitor alpha | N/A |
| quercetin | NADPH--cytochrome P450 reductase | DrugBank |
| quercetin | Ornithine decarboxylase | DrugBank |
| quercetin | Xanthine dehydrogenase/oxidase | DrugBank |
| quercetin | Caspase-8 | N/A |
| quercetin | DNA topoisomerase 1 | DrugBank |
| quercetin | RAF proto-oncogene serine/threonine-protein kinase | N/A |
| quercetin | Superoxide dismutase [Cu-Zn] | DrugBank |
| quercetin | Protein kinase C alpha type | N/A |
| quercetin | Interstitial collagenase | DrugBank |
| quercetin | Hypoxia-inducible factor 1-alpha | N/A |
| quercetin | Signal transducer and activator of transcription 1-alpha/beta | N/A |
| quercetin | Protein CBFA2T1 | N/A |
| quercetin | Probable E3 ubiquitin-protein ligase HERC5 | N/A |
| quercetin | Cell division control protein 2 homolog | DrugBank |
| quercetin | 78 kDa glucose-regulated protein | DrugBank |
| quercetin | Receptor tyrosine-protein kinase erbB-2 | N/A |
| quercetin | Peroxisome proliferator-activated receptor gamma | N/A |
| quercetin | Acetyl-CoA carboxylase 1 | DrugBank |
| quercetin | Heme oxygenase 1 | DrugBank |
| quercetin | Cytochrome P450 3A4 | DrugBank |
| quercetin | Cytochrome P450 1A2 | DrugBank |
| quercetin | Caveolin-1 | N/A |
| quercetin | Myc proto-oncogene protein | N/A |
| quercetin | Glutathione S-transferase Mu 2 | DrugBank |
| quercetin | Gap junction alpha-1 protein | DrugBank |
| quercetin | Cytochrome P450 1A1 | N/A |
| quercetin | Intercellular adhesion molecule 1 | N/A |
| quercetin | Interleukin-1 beta | DrugBank |
| quercetin | C-C motif chemokine 2 | DrugBank |
| quercetin | E-selectin | DrugBank |
| quercetin | Vascular cell adhesion protein 1 | DrugBank |
| quercetin | Prostaglandin E2 receptor EP3 subtype | DrugBank |
| quercetin | Interleukin-8 | N/A |
| quercetin | Protein kinase C beta type | N/A |
| quercetin | Baculoviral IAP repeat-containing protein 5 | N/A |
| quercetin | Dual oxidase 2 | N/A |
| quercetin | Nitric oxide synthase, endothelial | N/A |
| quercetin | Heat shock protein beta-1 | N/A |
| quercetin | Transforming growth factor beta-1 | N/A |
| quercetin | Estrogen sulfotransferase | DrugBank |
| quercetin | Maltase-glucoamylase, intestinal | DrugBank |
| quercetin | Interleukin-2 | DrugBank |
| quercetin | Nuclear receptor subfamily 1 group I member 2 | N/A |
| quercetin | Cytochrome P450 1B1 | N/A |
| quercetin | G2/mitotic-specific cyclin-B1 | N/A |
| quercetin | Tissue-type plasminogen activator | DrugBank |
| quercetin | Thrombomodulin | DrugBank |
| quercetin | Plasminogen activator inhibitor 1 | N/A |
| quercetin | Collagen alpha-1(I) chain | DrugBank |
| quercetin | Interferon gamma | DrugBank |
| quercetin | Arachidonate 5-lipoxygenase | DrugBank |
| quercetin | Phosphatidylinositol-3,4,5-trisphosphate 3-phosphatase and dual-specificity protein phosphatase PTEN | N/A |
| quercetin | Interleukin-1 alpha | N/A |
| quercetin | Myeloperoxidase | DrugBank |
| quercetin | DNA topoisomerase 2-alpha | N/A |
| quercetin | Neutrophil cytosol factor 1 | N/A |
| quercetin | ATP-binding cassette sub-family G member 2 | N/A |
| quercetin | Hyaluronan synthase 2 | N/A |
| quercetin | Glutathione S-transferase P | DrugBank |
| quercetin | Nuclear factor erythroid 2-related factor 2 | N/A |
| quercetin | NAD(P)H dehydrogenase [quinone] 1 | DrugBank |
| quercetin | Poly [ADP-ribose] polymerase 1 | N/A |
| quercetin | Aryl hydrocarbon receptor | DrugBank |
| quercetin | 26S proteasome non-ATPase regulatory subunit 3 | N/A |
| quercetin | Solute carrier family 2, facilitated glucose transporter member 4 | N/A |
| quercetin | Collagen alpha-1(III) chain | DrugBank |
| quercetin | DNA gyrase subunit B | DrugBank |
| quercetin | C-X-C motif chemokine 11 | N/A |
| quercetin | C-X-C motif chemokine 2 | N/A |
| quercetin | DDB1- and CUL4-associated factor 5 | N/A |
| quercetin | Nuclear receptor subfamily 1 group I member 3 | N/A |
| quercetin | Serine/threonine-protein kinase Chk2 | N/A |
| quercetin | Insulin receptor | DrugBank |
| quercetin | Claudin-4 | N/A |
| quercetin | Peroxisome proliferator-activated receptor alpha | N/A |
| quercetin | Peroxisome proliferator-activated receptor delta | N/A |
| quercetin | Heat shock factor protein 1 | N/A |
| quercetin | C-reactive protein | N/A |
| quercetin | C-X-C motif chemokine 10 | N/A |
| quercetin | Inhibitor of nuclear factor kappa-B kinase subunit alpha | N/A |
| quercetin | Osteopontin | N/A |
| quercetin | Runt-related transcription factor 2 | N/A |
| quercetin | Ras association domain-containing protein 1 | N/A |
| quercetin | Transcription factor E2F1 | N/A |
| quercetin | Transcription factor E2F2 | N/A |
| quercetin | Prostatic acid phosphatase | DrugBank |
| quercetin | Cathepsin D | DrugBank |
| quercetin | Insulin-like growth factor-binding protein 3 | N/A |
| quercetin | Insulin-like growth factor II | N/A |
| quercetin | CD40 ligand | N/A |
| quercetin | Interferon regulatory factor 1 | N/A |
| quercetin | Receptor tyrosine-protein kinase erbB-3 | N/A |
| quercetin | Serum paraoxonase/arylesterase 1 | DrugBank |
| quercetin | Type I iodothyronine deiodinase | N/A |
| quercetin | Procollagen C-endopeptidase enhancer 1 | N/A |
| quercetin | Puromycin-sensitive aminopeptidase | N/A |
| quercetin | Hexokinase-2 | N/A |
| quercetin | Homeobox protein Nkx-3.1 | N/A |
| quercetin | Ras GTPase-activating protein 1 | N/A |
| quercetin | Peroxidase C1A | N/A |
| quercetin | Glutathione S-transferase Mu 1 | DrugBank |
| quercetin | Tissue factor | DrugBank |
